# Annotations

**Published:** 1901-11-02

**Authors:** 


### Annotations.

### Lepers in England.

We do not wish to enter into the perennial controversy in regard to the constant flow of undesirable aliens towards the ever hospitable shores of England. We may, however, point to a concrete instance which serves to show how free trade in undesirables, as exemplified in English practice, is likely to work out when we are surrounded by a ring fence of nations, all of them provided with laws, such as have lately been passed in the United States, by which they are protected from the ingress of those who are morally or physically unfitted to make useful citizens. In the recently-issued Report of the medical officer for the Port of London, Dr. Collingridge describes how he received information from the owners of the sailing ship *Fingal* that one of the crew of that vessel shipped at Calcutta, was found to be a leper, and that they had been forbidden by the United States Government to land the man at San Francisco. On the vessel's arrival at Gravesend "the patient was removed to the West London Hospital at the expense of the owners, this disease not being one dealt with under the Public Health Act." It is not to be expected, however, that the owners will make themselves permanently chargeable with this patient, so here we have another "undesirable" dumped upon our shores. Whether or not, coming from Calcutta, he is a fellow subject, or merely a casual picked up at that cosmopolitan port of call, we do not know. The point is that while other countries refuse to receive these people we admit them without question. It is not difficult to guess what would be the position of a shop-keeper who raised no question about false money, while his competitors rang every penny on the counter and refused to accept any but current coin of the realm. He would no doubt for a time seem to do a roaring trade, and undoubtedly would be a very popular person in shady circles, but he certainly before long would go to smash. Let us take care that we do not follow his example.

### Our Friends the Quacks.

Among the delights of living in a free country is the liberty of being humbugged to one's heart's content which one there enjoys. This is indeed one among the many bonds between England and America, for at whichever side of the Atlantic one resides the quacks are always with us, and however dull the newspapers may be, one can always turn to the amusing lies of those who promise to cure us of all diseases. So strange to the American does any interference with the lying advertisements of quack medicines appear, that the United States Consul at Vienna has sent in a report showing, for the edification of his fellow countrymen, the measures adopted in Austria for the protection of the people from the wiles of the advertiser. From this document it appears that the sale of secret remedies is there forbidden, and that no apothecary is permitted to keep for such purpose any medicinal preparation the formula of which is not open to inspection by physicians, or "in the prescription of which the substance of the medicinal ingredients cannot be definitely recognised as to kind and quantity." Moreover Government inspection of all new preparations is required before their sale is allowed, and a rule exists absolutely prohibiting, on the labels, wrappers or advertisements of any cosmetics, any claim that they are efficacious in removing personal blemishes. Another serious blow to the quack advertiser exists in the fact that no testimonials from abroad of the cures effected by patent remedies may be published in the local papers. To some, no doubt, all this will seem to be a serious interference with personal liberty; to many, we hope, it will appear a reasonable endeavour to enforce honesty of dealing and to put a stop to false pretences. The Anglo-Saxon race, however, loves to be quacked, and honours the man who can puff his wares with the greatest impudence.

### Medical Reform.

In his speech at the Birmingham University on October 24th, Mr. Victor Horsley stated that there were three great needs of the profession. They were not satisfied with the *status* of their profession as compared with other learned professions; the relations of it to the public were strained, and there was a need of a rearrangement of its internal government. He might perhaps have added that the social status of the medical profession obviously rests upon that of its members primarily, and secondly upon the fact that no State appointments of great importance are open to them. A barrister may become a judge, a clergyman a bishop, both of which positions command a certain social status, but with the exception of these a successful medical man of manners and education takes the same position as any other professional man. As to the public, they are becoming sceptical in every way, and wish now to decide for themselves questions of science, just as they have long since insisted upon formulating their own religious and political opinions. They think they have a right to know the why and wherefore of the treatment recommended, and one way of removing the "strained" relationship is to endeavour to explain in simple language to all who wish to learn the general principles and methods which underlie medical work. The term "trade union" as applied in a reproachful way to the medical profession, is, of course, a ridiculous misnomer. It means that a body of men have joined together to defend themselves and to make war upon the public, and its application to the medical profession is due to an entirely false conception of all higher aims and objects of medical science. The only way to treat such an accusation is by a dignified and contemptuous silence, trusting that in time the fact will be brought home that in reality the greater part and energy of every practitioner's life is devoted to the public good and not to private gain. Unfortunately, the *esprit de corps* of the medical profession as a whole is not as strong as it ought to be, or used to be, and the life of each member is so occupied with his own particular practice or line of research that he has no clear idea of acting as an unit of a great body. It is useless looking to the central government unless some definite propositions are sent unanimously, and we fear that is a long way off. By the time we are unanimous perhaps the general Medical Council may have become so reformed as to really represent the bulk of practitioners and give voice to their demands.

